# The stepwise implementation of a safe and effective Endoscopist-Directed Nurse-Administered Propofol Sedation model of care in Queensland for low-risk endoscopic procedures

**DOI:** 10.3389/frhs.2025.1666181

**Published:** 2025-12-04

**Authors:** Ameya Godambe, Rhian Jones, Virginia DeCourcy, Kate Bennett, Angela Carberry, James O’Beirne

**Affiliations:** 1 Sunshine Coast University Hospital, Birtinya, QLD, Australia; 2 Sunshine Coast Health Institute, Birtinya, QLD, Australia; 3 Griffith University, Gold Coast, QLD, Australia

**Keywords:** colonoscopy, sedation, propofol, safety, endoscopy

## Abstract

**Introduction:**

Current models of gastrointestinal endoscopy provision in Queensland are heavily reliant on the use of specialist anaesthetists to deliver sedation. We evaluated the implementation of Endoscopist-Directed Nurse-Administered Propofol Sedation (EDNAPS) as a model of care for low-risk colonoscopy procedures in Queensland.

**Method:**

Staff were recruited and trained using a combination of face-to-face sessions, self-directed learning and high-fidelity simulation. 118 colonoscopies were performed using EDNAPS and compared to 118 procedures with anaesthetist-delivered sedation (comparison group). A mixed-methods approach was used to collect data regarding the timing, safety and adenoma detection rate, as well as obtaining survey responses from patients and clinicians on the safety and effectiveness.

**Results:**

Our study found no statistically significant difference in safety or clinical outcomes between the two groups (EDNAPS vs. comparison). However, the time spent in the Post-Anaesthesia Care Unit (PACU) post-endoscopy was significantly shorter in the EDNAPS group (*p* < 0.01). 48 Patient Survey responses obtained (21% response rate) were largely in favour of the safety and effectiveness of EDNAPS.

**Conclusion:**

The findings of our study not only re-affirm the safety and effectiveness of EDNAPS, but also provide a locally endorsed implementation model which can aid in the wider adoption of this practice, statewide and nationally.

## 1 Introduction

There is increasing demand for healthcare resources in Queensland's Public Health system. This can at least in part be attributed to a rising population, decreased utilisation of the private sector and a shortage of qualified staff (1). The Queensland Government estimates a need for 19,000 more nurses and 6,000 more doctors by 2032 to meet its population's healthcare needs (1, 2). As such, it has become increasingly important to identify traditional medical practices which can be adapted to reduce the burden on a growing health service whilst maintaining patient outcomes, safety and satisfaction. One such procedure open to adaptation is endoscopy.

Gastrointestinal Endoscopy (GIE) is a common procedure performed for diagnostic and therapeutic purposes. In Australia, sedation for GIE procedures is traditionally provided by anaesthetists using propofol combined with opiates and benzodiazepines. Propofol is an anaesthetic agent and as such has required anaesthetist support to administer, monitor the patient and manage any adverse sedation related events (3). However, evidence suggests that complications related to propofol-based sedation are rare outside of patients pre-identified to be at high risk (4). In light of this, alternative models of endoscopy sedation have been developed. One such alternative model involves the administration of propofol sedation by a trained nurse directed by the endoscopist, so-called Endoscopist-Directed Nurse-Administered Propofol Sedation (EDNAPS) (3). Proceduralist-led sedation and particularly the EDNAPS model has been safely carried out in a range of hospital settings nationally and internationally, although uptake in Australia has been limited to only a few centres (3, 5–10). The safety and efficiency of the EDNAPS model for both upper and lower GI endoscopy has recently been demonstrated in a report from Cairns Hospital where 25,000 procedures were performed under the EDNAPS model with a sedation related complication rate of only 0.26% (3).

Flexible models of endoscopy sedation such as EDNAPS offer several potential benefits including reduced procedural cost, increased procedural access and it releases anaesthetists to be available in other clinical settings (7). While substantial literature supports the use of EDNAPS, the introduction of new care models into novel healthcare settings, such as our health service, necessitates careful implementation. Demonstrating local efficacy and safety is critical to achieving successful adoption and stakeholder acceptance. This study aimed to assess the implementation, effectiveness, safety, and patient satisfaction associated with the EDNAPS model of care for low-risk patients in a Queensland hospital setting.

## 2 Method

### 2.1 The EDNAPS model of care

#### 2.1.1 Setting

The evaluation was conducted at the Sunshine Coast University Hospital (SCUH) and Nambour General Hospital (NGH), two sites within the Sunshine Coast Hospital and Health Service (SCHHS) in South-East Queensland, Australia, between 1 August 2023 and 31 July 2024.

The SCHHS delivers over 10,000 endoscopic procedures per year in five endoscopy rooms over two sites. SCUH has three rooms and mainly deals with acute and interventional patients, with two further rooms at NGH accommodating routine diagnostic cases. The SCHHS has a catchment area of approximately 460,000 people (11, 12).

#### 2.1.2 Staff training

Four nursing staff were recruited via an open merit Expression of Interest (EOI) process within SCHHS to be part of the EDNAPS program. All staff, including nursing and medical officers, completed required training which included Intravenous Cannulation (IVC) insertion and Advanced Cardiovascular Life Support (ACLS). The EDNAPS staff underwent a credentialling process, which included self-directed learning and attendance at face-to-face training workshops (Medical Officers completed 16 h and Registered Nurses completed 22 h). The workshops consisted of didactic lectures on pharmacology, airway assessment, monitoring and anaesthetic emergencies followed by high fidelity simulation exercises. All staff working in the model attended a two-day workshop including summative assessment at Cairns Hospital and Health Service, where an established EDNAPS program has been in place for ten years (3). A preceptorship program was established with a staged approach over 30 colonoscopies. Preceptees, who were either Anaesthetists or Medical Officer Sedation Champions, initially provided direct supervision to staff, which reduced to indirect supervision over time. Final sign off was provided by the Head of Anaesthetics and Senior Medical Officer in Gastroenterology.

#### 2.1.3 Pre-implementation planning process

In preparation for implementing EDNAPS into clinical practice at SCHHS, the multidisciplinary project team collaborated to identify the requirements to support a new model of care. This included face-to-face and virtual meetings, written and verbal communication relating to clinical guidelines, formal training requirements, credentialing and simulation testing. A desktop process mapping exercise was conducted to test the end-to-end process from administration, referral, procedure and final handover to the recovery team. A formative gap analysis was conducted, and all required actions were resolved. A subsequent simulation session was held in the high-fidelity simulation suites at the Sunshine Coast Health Institute (SCHI) at SCUH. Pre-theatre protocols, Business as Usual (BAU) endoscopy procedures and patient deterioration were tested. Agreement was reached by all key stakeholders that the model of care had been tested and accepted as service-ready prior to the service going live.

#### 2.1.4 The EDNAPS procedure

Patients referred to SCHHS for colonoscopy were identified as potential candidates for EDNAPS if they were American Society of Anesthesiologists' (ASA) Physical Status Classification score of I or II, aged 75 years or below and had a body mass index of less than 35 kg/m^2^. Patients attending procedures predicted to be prolonged (resection of large polyps, polyposis syndrome surveillance) were excluded.

Patients were consented for the procedure as per Queensland Health guidelines and underwent brief assessment by the endoscopist and EDNAPS nurse focusing on airway assessment, anaesthetic history and vital signs prior to the procedure (13). All patients underwent continuous cardiac monitoring, cycled non-invasive blood pressure monitoring and capnography. All patients received high flow nasal oxygen supplementation.

Initial sedation consisted of midazolam [1–3 mg intravenous (IV)] and propofol (10–50 mg IV) administered by the endoscopist. After reaching the desired level of sedation the procedure commenced and further aliquots of propofol (10–20 mg IV) were administered by the EDNAPS nurse under the direction of the endoscopist.

All EDNAPS procedures were performed by the same endoscopist. Due to the pilot nature of the study and the need for off-site training it was deemed to be impractical to provide training to multiple endoscopists.

### 2.2 Data analysis

To evaluate the effectiveness of the EDNAPS model, a mixed methods approach was adopted incorporating both quantitative and qualitative analysis.

#### 2.2.1 Quantitative analysis

##### 2.2.1.1 Design

A retrospective comparative analysis was undertaken to compare the characteristics and outcomes of patients who underwent an EDNAPS procedure with those of patients who underwent a standard endoscopy, performed by various endoscopists within the department. Eligible participants were those undergoing endoscopy with an ASA Physical Status Classification score of I or II on days when EDNAPS procedures were taking place (14).

All patients who had undergone an EDNAPS procedure were included. The comparison group were selected from patients who had undergone endoscopy with anaesthetist sedation and were matched 1:1 to EDNAPS patients on date and facility in order to control for other factors such as staffing levels. Patients were selected based on ASA score and age. Co-morbidities and BMI were not matched for as these factors were felt to have been adequately excluded from both groups, noting that co-morbidities significantly affecting sedation risk would have scored an ASA of 3 or greater.

##### 2.2.1.2 Variables

The exposure variable was whether patients received sedation administered under the EDNAPS model or by an anaesthetist (comparison group). Outcome variables were colonoscopy withdrawal time, total time elapsed in the procedure, time elapsed between admission to and discharge from the Post-Anaesthesia Care Unit (PACU), and whether adenoma or sessile serrated polyps were detected during the procedure. Patient background information was collected including ASA score, age in years and gender. A range of indicator variables for patient safety were also recorded (in-procedure complications; post-procedure presentation to the Emergency Department; post-procedure hospital admission; and death). Safety data was collected for each patient by examining procedural documentation (endoscopy reports and anaesthetic reports for each patient). In order to identify post-procedural complications, we examined the iEMR (integrated electronic medical record) for each patient for 72 h post-procedure, in addition to a statewide electronic admissions system (The Viewer) to identify admissions to other health facilities within the 72 h post-procedure. The outcomes we were looking to assess procedural safety included any report of intra-procedural complications due to the endoscopy or anaesthesia, any hospital presentations or admissions within 72 h of the procedure and any patient deaths within 72 h of the procedure.

##### 2.2.1.3 Statistical analysis

Patient data was extracted from the Integrated Electronic Medical Record (ieMR—the SCHHS medical records system), Cerner (now known as Oracle Health, an internationally developed digital healthcare platform) and Provation MD (endoscopy reporting software) and stored in a secure spreadsheet. Descriptive analysis was conducted to determine frequencies of demographic and patient safety characteristics across the two groups. Outcome variables were compared between groups, with Mann–Whitney *U* tests used to determine statistical significance (*p* < 0.05) for continuous variables and chi-squared tests for categorical variables. Linear regressions were conducted for each outcome to control for the effect of age and ASA. Analyses were conducted in jamovi version 2.5. *P* < 0.05 was the level of significance for two tailed tests.

#### 2.2.2 Qualitative analysis

##### 2.2.2.1 Design

Qualitative information was collected to gain understanding of the patient and clinician experience using EDNAPS. Thematic analysis was used to analyse, code and interpret narrative responses and subsequently identify recurring patterns in participants' responses. Eligible participants were identified as patients included in the quantitative analysis, SCHHS staff members who participated in the EDNAPS training, implementation and delivery, and SCHHS staff members who worked in the Gastroenterology and Anaesthesiology departments during the study period.

##### 2.2.2.2 Clinician surveys

Two online surveys were conducted three months post-implementation of EDNAPS, with the purpose of examining staff and clinician experience when accessing, providing and supporting EDNAPS. Both surveys included a combination of Likert scale responses and open-ended free-text questions. The two surveys are described below:

- EDNAPS Translational Simulation Survey—provided to all SCHHS staff members who participated in the online education and high-fidelity simulation prior to implementing EDNAPS.
- EDNAPS Experience Survey—provided to all staff members within the Gastroenterology and Anaesthesiology departments, irrespective of their involvement in implementing EDNAPS.

##### 2.2.2.3 Patient survey

An online consumer anonymous survey was sent to all EDNAPS and comparison patients post-procedure using a secure one-way messaging service. All responses remained anonymous, and access to data was restricted to authorised Queensland Health staff. Written consent was obtained from all participants, outlining that their participation was voluntary, and their information would be handled in a confidential manner.

### 2.3 Ethics

This evaluation was classified as a quality improvement initiative and thus an ethics exemption was granted from Gold Coast HHS Human Research Ethics Committee (HREC)—EX/2023/QGC/105011.

## 3 Results

### 3.1 Quantitative analysis

Between 1 August 2023 and 30 July 2024, 120 patients underwent an EDNAPS procedure. 118 of these were included in the quantitative analysis, with two excluded because they did not have a recorded ASA score of I or II. After matching, 118 patients who underwent standard endoscopy were included in the comparison group.

#### 3.1.1 Descriptive analysis

Table 1 presents key characteristics of the two groups.

**Table 1:** Demographic and clinical characteristics of participants in the EDNAPS and comparison group.

Demographic	EDNAPS group (*n* = 118)	Comparison group (*n* = 118)	Difference between groups
Age (median, years)	53.0	57.5	*p* < 0.01
Male	58 (49.2%)	58 (49.2%)	*p* = 1.00
Female	60 (50.8%)	60 (50.8%)	
ASA score I	39 (33.1%)	19 (16.1%)	*p* < 0.01
ASA score II	79 (66.9%)	99 (83.9%)	
Procedure performed at Sunshine Coast University Hospital	41 (34.7%)	42 (35.6%)	*p* = 0.89
Procedure performed at Nambour General Hospital	77 (65.3%)	76 (64.4%)	

As per the matching criteria, procedure facility had the same distribution across groups, as did gender, although it was not a matching criterion. The median age in the EDNAPS group (53 years) was significantly lower than the comparison group (57.5 years), as was ASA score, with 84% of the comparison group having a risk score of II compared to 67% of the EDNAPS group.

For patient safety variables, there was one report of in-procedure complications, one post-procedure hospital admission, and one emergency department presentation, all among the comparison group and occurring for the same patient. There were no deaths in either group.

#### 3.1.2 Comparative analysis

Table 2 presents the results of the comparative analysis.

**Table 2:** Outcome variables in the EDNAPS and comparison groups.

Outcome	EDNAPS group (*n* = 118)	Comparison group (*n* = 118)	*P*-value (univariate)	*P*-value (multivariate)
Median scope withdrawal time (mins)	12.7	13.7	0.06	0.01
Median total procedure time (mins)	21.7	21.5	0.99	0.20
Median time in Post-Anaesthesia Care Unit (mins)	18.0	24.0	<0.01	<0.01
Adenoma detected (%)	48.3	51.7	0.60	0.66
Sessile lesion detected (%)	17.8	28.8	0.05	0.07

At univariate analysis, there was no significant difference between the EDNAPS and comparison groups in terms of scope withdrawal time or total procedure time, and no significant difference in the detection of adenoma or sessile lesions. However, time spent in PACU was significantly shorter among EDNAPS patients than the comparison group, with median times of 18 and 24 min respectively.

After controlling for age and ASA score, there remained no difference between groups for total procedure time or polyp detection, and time in PACU remained significantly lower among EDNAPS patients. The difference in scope removal time reached statistical significance at multivariate analysis (*p* = 0.01), although it was not clinically significant (median difference of 1.0 min).

Table 3 presents a summary of the intra- and post-procedural safety data collected.

**Table 3:** Patient safety data.

Safety data	EDNAPS group (*n* = 118)	Comparison group (*n* = 118)
Intra-procedural complications	0	0
Post-procedural hospital presentations and/or admissions (within 72 h)	0	1
Post-procedural mortality (within 72 h)	0	0

### 3.2 Qualitative analysis

#### 3.2.1 Patient survey

The survey received 48 responses (21% response rate) comprising 22 EDNAPS patients and 26 from the standard care comparison group. Table 4 presents an overview of responses to quantitative questions in the survey.

**Table 4:** Quantitative data from survey responses by the EDNAPS and comparison groups.

Survey responses	EDNAPS group (*n* = 22)	Comparison group (*n* = 26)
Overall rating: Very good/Good	21 (95%)	25 (96%)
Overall rating: Adequate	0	0
Overall rating: Poor/Very Poor	1 (5%)	1 (4%)
Staff explained care in a way I understood	22 (100%)	26 (100%)
I was involved in care decisions	21 (95%)	25 (96%)
I experienced discomfort during the procedure	2 (9%)	0
I had an opportunity to ask about findings	19 (86%)	23 (88%)

Overall, patients in both groups were highly satisfied with their care, felt that staff explained care in a way they could understand, and felt that they were involved in care decisions and were able to ask about findings after the procedure. While most patients did not report experiencing any discomfort, two EDNAPS patients reported discomfort during the procedure and four after the procedure, which was higher than reported discomfort in the comparison group.

EDNAPS patients reported a range of feedback in free text responses related to their experience and suggestions for improvement. The professionalism and kindness of healthcare staff was strongly emphasised, and respondents reported feeling “at ease”, “safe”, and “informed”. Quotes to further illustrate patient satisfaction with the process included the following:

“All staff were very professional, caring and understanding, answering and reassuring any concerns I had.”

“Every person was kind, lovely and I felt I was in a safe place. The doctor was exceptional, making me feel at ease and well cared for.”

While most EDNAPS patients reported feeling highly satisfied with their experience, two patients complained of pain, including one who reported pain from the cannula being inserted, and another who reported experiencing pain during the procedure and not feeling as though they were “given enough drugs to knock me out”.

#### 3.2.2 Clinician surveys

##### 3.2.2.1 EDNAPS translational simulation survey

A total of eight (*n* = 8) responses were received (62% recruitment rate) across a range of professional streams, including nursing, medical and administrative staff.

Overall, respondents reported satisfaction with the translational simulation and found its inclusion beneficial to their training and subsequent clinical practice. Analysis of the free-text responses revealed some positive key themes, with the translational simulation being described as a valuable tool for reinforcing skills, improving staff confidence, identifying potential complications ahead of time and fostering a sense of teamwork and collaboration. Responses that further exemplified this included the following:

“Hands on situational training. Teams working closely together, training together, in a timely fashion.”

“It reinforced to the staff involved that they possessed the necessary skills and knowledge to safely manage patients undergoing colonoscopy using the EDNAPS process.”

Notably, while the responses for the high-fidelity simulation were largely positive, feedback about the desktop simulation was more varied, with some respondents finding its duration too long and its content difficult to follow. Other suggestions for improvement included the addition of a training component for the administrative staff, so they can better understand the EDNAPS model of care in order to support the department and field questions from patients, as well as increasing the provision of EDNAPS so that staff in training have suitable opportunity to attend supervised lists and obtain credentials to formally practice with EDNAPS.

##### 3.2.2.2 Clinician experience survey

A total of 15 responses (unable to determine recruitment rate due to survey distribution method) were received from staff across a range of professional streams, primarily within the Gastroenterology department (80%). 80% of respondents were aware that the EDNAPS model of care was being implemented in the Health Service. Most respondents (60%) felt that EDNAPS was a safe model of care and added value to practices within the Gastroenterology (67%) and Anaesthetic (60%) departments.

With regards to points in favour of the long-term implementation of EDNAPS, respondents commented on the improved recovery post-procedure, resulting in a quicker turnaround and more accessibility. Respondents also fed back about the supportive and enthusiastic nature of EDNAPS staff members, as well as reiterating the strong sense of teamwork and good communication experienced by those working with the EDNAPS model of care.

Respondents raised concerns about the long-term implementation of EDNAPS, including management of rare but potential airway emergencies, limited involvement by the Anaesthetics team in the formation of the EDNAPS model of care, and the limited availability of EDNAPS lists, thus making training and credentialling of staff more challenging. 47% stated they would like to be involved in EDNAPS in the future, stating that it appears to be a safe and accessible alternative to the current model of care, with exciting career development opportunities for the staff involved. A further 33% stated they were “Unsure”, citing that they needed more training, in areas such as airway management, as well as more information, including more evidence to support that EDNAPS is more efficient. The remaining 20% “Did not wish to participate” in EDNAPS in the future, stating that they “did not want the responsibility” and did not feel comfortable performing the procedure without an anaesthetist present.

## 4 Discussion

The most valuable insights offered by our study come from the practical steps required to implement the EDNAPS model in a novel clinical setting. Although only a small pilot study, we have outlined a structured approach to delivery which may serve as a framework for scaling EDNAPS across similar health services—potentially improving overall efficiency and access. In addition, this project adds to the growing body of evidence in support of the safety and effectiveness of EDNAPS, focusing on colonoscopy in low-risk patients (ASA score of I or II). There was no significant difference in safety with no reports of any intra-procedural complications, post-procedure hospital admissions within 72 h or procedure-associated mortality in the EDNAPS cohort (1 patient in the comparison group required a post-procedural medical assessment and admission). Survey results by patients and staff revealed no further adverse events and most responses described EDNAPS as an appropriately safe model, except for a few concerns about potential airway difficulties, for which the high-fidelity simulation was implemented, helping nursing staff to prepare for and minimise this risk. The effectiveness of the procedure was also maintained with EDNAPS, with no significant difference in adenoma detection rate or total procedure time between the two groups. The findings of our study are in keeping with a growing body of evidence on the safety of non-anaesthesiologist administered propofol (NAAP) sedation in endoscopy, a category into which EDNAPS falls (14–16). Multiple reports are available worldwide showing that there is no added safety risk or poorer procedure outcome with EDNAPS and related practices (3, 5–7, 15–19). Several large-scale studies have also been performed, the largest of which looked at over 1.3 million endoscopy cases using EDNAPS, arriving at the conclusion that EDNAPS carries no added safety risk when compared to anaesthetist-led sedation (3, 5, 9, 17). In fact, some studies found a higher adverse event risk in groups sedated by anaesthetists vs. non-anaesthetists, possibly due to longer periods of deep sedation which may increase the risk of cardiopulmonary events (5, 9). Outside of Australia, nurse-led or non-anaesthesiologist sedation is becoming increasingly common for endoscopy, reflected in its inclusion in several guidelines (8, 20). EDNAPS as a model of care is emerging as a viable option for endoscopy delivery in Australia. Cairns Hospital recently published their experience of over 25,000 endoscopies using EDNAPS and their results demonstrated that EDNAPS is safe and effective, particularly in patients with an ASA score of I or II (3). Importantly, alongside maintaining an appropriate standard of patient safety, evidence shows that the adenoma detection rate is equivalent in colonoscopies performed using NAAP vs. those with anaesthetist-led sedation, preserving the clinical utility of the procedure (21).

Aside from adequate patient safety, our research shows that EDNAPS may provide several further benefits. Patients in the EDNAPS cohort spent a significantly shorter time in PACU (Post-Anaesthesia Care Unit) following the procedure, suggesting a faster recovery. This is likely due to a reduced total dose of propofol during the procedure. In EDNAPS patients, propofol was administered under the direction of the endoscopist based on patient comfort, as reported by the patient, EDNAPS nurse, or perceived by the endoscopist. In the comparison group, propofol boluses were used under the anaesthetist's direction or given by infusion, targeting a specific blood concentration, which likely resulted in higher cumulative propofol doses being delivered.

Several studies have found propofol use, compared to benzodiazepines, can result in a quicker return to baseline psychomotor function, likely due to its short half-life as it undergoes rapid hepatic clearance (18, 22, 23). Propofol can be used as a single agent or in combination with opioids, where it has been reported to provide better patient comfort and satisfaction, as well as a deeper degree of sedation when compared to midazolam with opioids (9, 18, 22). A systematic review examining numerous sedative agents found that midazolam also carries a slightly higher risk of memory difficulty post-procedure when compared to propofol (23). While there may have been historic concerns about the narrow therapeutic window and lack of reversal agent with propofol use, research has shown that it can be safely administered by trained nurses and non-anaesthesiologists (3, 5, 7, 9, 15–19). Propofol is currently a more expensive medication, but this can be offset by an overall reduced procedural cost when using trained nurses for sedation rather than anaesthesiologists (18).

EDNAPS has the potential to be a more cost-effective model of care for two important reasons. Specially trained nurses can be used, whose average predicted salary is less than that of anaesthetists in the same role (24, 25). Based on current models, it is predicted that there will be a 5.7% shortfall by 2032 between the available anaesthetic workforce and that which is required in Australia (26). The implementation of EDNAPS may enable better reallocation of anaesthetist services to minimise this shortfall. Our research and that of others has also demonstrated that EDNAPS results in a faster recovery post-procedure. The cost implications of this finding are that more procedures can be performed within a given time frame if EDNAPS is used, and there can be more efficient use of recovery spaces such as PACU. This cost benefit could have huge implications when considering the large-scale use of colonoscopies as part of national bowel cancer screening programs. In the United States, one analysis predicted a 10-year saving of 3.2 billion US dollars with endoscopist-directed propofol sedation rather than traditional anaesthesiologist-led sedation (25). While it can be difficult to accurately provide an exact figure, early analysis demonstrates that EDNAPS may allow for more effective use of healthcare resources. The recent reduction in the age of entry into the national bowel cancer screening program to 45 years will increase demand for colonoscopy and this group would be particularly suited for flexible models of endoscopy care such as EDNAPS (27).

Our study had several limitations. One important limitation is the use of one endoscopist for all the study group patients, while multiple endoscopists were used in the comparison group, introducing operator bias. Evidence shows that there can be significant inter-operator variability in terms of clinical outcomes, such as adenoma detection rate, as well as complication rates (28–31). While we were limited to one endoscopist due to the pilot nature of this study, future studies would preferably include multiple endoscopists, which will be made more achievable once EDNAPS is a better-established practice. Of interest is the evidence highlighting inter-operator variability in sedation use, which would be interesting to evaluate in future projects incorporating data on sedation depth/dosage (32). In addition, this study was conducted in only two hospitals within Queensland, limiting its external validity, and future studies should aim to implement this as part of a wider, multi-centre operation, in order to evaluate its use in different settings and better justify its state-wide application.

Another limitation is the inability to match patient and comparisons groups for ASA class, with a higher proportion of ASA 1 patients seen in the study group. Whilst this may have influenced outcomes, in practice, we anticipate the effect to be relatively small. The biggest indicators of cardiopulmonary risk from sedation in endoscopy are advanced age (which was matched) and BMI >40 (we excluded patients with a BMI >35) (33, 34). Whilst ASA class is a useful predictor of sedation risk, data shows that the risk does not increase linearly with ASA class (33, 35). The relative difference in risk between ASA class 1 and 2 is small, and rises significantly with classes 3 and greater, which were again excluded from our study (33, 35). A major limitation of the study is that we were unable to directly compare propofol use between the two groups as most procedures in the comparison group used target-controlled propofol infusions. In these cases, the target concentration was recorded in the medical record rather than total propofol dose. However, as touched upon earlier, anaesthetist-led sedation has been associated with higher doses of sedative medication, which may be a factor in the increased time spent in PACU post-procedure (5, 9). There is scope for more research, with potential utilisation of validated sedation monitoring tools, such as the Richmond Agitation-Sedation Scale (RASS), to better standardise the approach.

A relatively small number of EDNAPS procedures were performed (*n* = 118), which restricted the complexity of the analysis and may have resulted in under-detection of adverse outcomes, as well as a relatively small sample of patients (21% response rate) and clinicians completing the online surveys, which therefore may not be fully representative of the evaluation cohort.

Areas for further research include more detailed examination of the potential cost-saving benefits of EDNAPS, as well as attempts to expand the breadth of EDNAPS to include higher risk patient cohorts and more challenging endoscopic procedures. Some research has already been conducted in this area, with promising evidence supporting its use in patient groups such those with obstructive sleep apnoea, as well as for procedures such as endoscopic ultrasound (15, 36).

## 5 Conclusion

The evaluation of the EDNAPS model of care at SCHHS demonstrated positive outcomes in terms of safety and effectiveness, but more notably, provides a framework for implementation which may play an important role in the wider adoption of EDNAPS across Queensland and Australia.

## Data Availability

The original contributions presented in the study are included in the article/Supplementary Material, further inquiries can be directed to the corresponding author.
